# Infant Appetitive Phenotypes: A Group-Based Multi-Trajectory Analysis

**DOI:** 10.3389/fnut.2021.749918

**Published:** 2021-12-24

**Authors:** Catherine G. Russell, Jessica Appleton, Alissa J. Burnett, Chris Rossiter, Cathrine Fowler, Elizabeth Denney-Wilson, Elena Jansen

**Affiliations:** ^1^School of Exercise and Nutrition Sciences, Institute for Physical Activity and Nutrition, Deakin University, Geelong, VIC, Australia; ^2^Susan Wakil School of Nursing and Midwifery, University of Sydney, Sydney, NSW, Australia; ^3^Tresillian Family Care Centres, Belmore, Sydney, NSW, Australia; ^4^Faculty of Health, School of Exercise and Nutrition Sciences, Deakin University, Melbourne, VIC, Australia; ^5^Faculty of Health, School of Nursing and Midwifery, University of Technology Sydney, Sydney, NSW, Australia; ^6^Sydney Local Health District, Sydney, NSW, Australia; ^7^Division of Child & Adolescent Psychiatry, Department of Psychiatry & Behavioral Sciences, Johns Hopkins University School of Medicine, Baltimore, MD, United States

**Keywords:** appetitive traits, appetitive phenotype, trajectories, infant, parent feeding, weight, appetite self-regulation, multi-trajectory analysis

## Abstract

**Background:** Examining appetitive traits with person-centered analytical approaches can advance the understanding of appetitive phenotype trajectories across infancy, their origins, and influences upon them. The objective of the present study was to empirically describe appetitive phenotype trajectories in infancy and examine the associations with infant and parent factors.

**Materials and Methods:** In this longitudinal cohort study of Australian infants, parents completed three online surveys ~3 months apart, beginning when the infant was <6 months. Appetitive traits were assessed with the Baby Eating Behavior Questionnaire (BEBQ) and parent feeding practices with the Feeding Practices and Structure Questionnaire (FPSQ) infant and toddler version. Parent demographics and cognitions were also collected. Infant weight and length were transcribed from health records and converted to a BMI *z*-score. Group-based trajectory modeling identified appetitive phenotype trajectories using the BEBQ. Multilevel modeling examined change in feeding practices and child BMI *z*-score over time by appetitive phenotype trajectories.

**Results:** At time 1, 380 participants completed the survey (mean infant age 98 days), 178 at time 2 (mean infant age 198 days), and 154 at time 3 (mean infant age 303 days). Three multi-trajectory appetitive phenotype groups were identified and labeled as (Phenotype 1) food avoidant trending toward low food approach (21.32% of infants), (Phenotype 2) persistently balanced (50.53% of infants), and (Phenotype 3) high and continuing food approach (28.16% of infants). Formula feeding was more common in Phenotype 1 (*p* = 0.016). Parents of infants in Phenotype 1 were more likely to rate them as being more difficult than average, compared to infants with phenotypes 2 or 3. Phenotype 2 had the greatest increase in persuasive feeding over time [0.30; 95% CI (0.12, −0.47)].

**Conclusions:** Distinct multi-trajectory appetitive phenotype groups emerge early in infancy. These trajectories appear to have origins in both infant and parent characteristics as well as parent behaviors and cognitions. The infant multi-trajectory appetitive phenotype groups suggest that for some infants, difficulties in self-regulating appetite emerge early in life. Investigation of infant multi-trajectory appetitive phenotype groups that utilize a range of measures, examine relationships to key covariates and outcomes, and extend from infancy into childhood are needed.

## Introduction

Children differ greatly in what and how they eat (1), with evidence that these differences emerge or are present in infancy (2, 3). These differences in children's early eating behaviors and attitudes have, in turn, been shown to contribute to later overweight and obesity in childhood (4). Appetitive traits are quantifiable individual differences in patterns of behaviors and attitudes related to food and eating (5, 6), typically measured with the parent-reported Baby Eating Behavior Questionnaire (BEBQ) for infants. Appetitive traits have been broadly classified into “food approach” (more avid appetite, greater interest in or desire to eat food) and “food avoidance” (lower appetite, lower interest in and desire to eat food) tendencies. Avid appetitive traits appear to make some individuals more susceptible to the effects of the obesogenic environment (e.g., the presence of food cues) and therefore greater weight gain (4, 7). Appetitive traits are highly heterogeneous, even in infancy (1–3) and while they are constitutionally based to varying degrees (3, 8), they are influenced over time by experiences (e.g., parent feeding practices) and development (9, 10).

The predominance of evidence about children's appetitive traits has focused on individual traits (such as fussiness or food responsiveness), with an analytic approach that is variable centered (4). Variable-centered approaches look at relationships between variables (e.g., satiety responsiveness and weight) to identify key variables and look at their prevalence and relationships to other variables in different groups (11). This approach is useful for answering research questions about the relative contributions of such variables to outcomes like weight gain. However, variable-centered approaches tend to use average scores on eating behavior variables and therefore do not examine individual differences or the presence of subgroups (12). A person-centered approach, in contrast, can answer research questions about individual and subgroup differences and looks for underlying latent variables that distinctively characterize different groups of children through underlying latent constructs. Latent profile or latent class analysis can be used with cross-sectional studies to assign individuals to a “profile” or “typology” based on patterns of relationships among particular variables of interest. This can lead to the identification of behavioral phenotypes or subgroups of children based on profiles of eating, that is, distinct patterns of behavior that arise due to a combination of genetic and environmental effects that impact health outcomes (5). Applying person-centered analytical approaches over time can lead to the identification of subgroups of individuals based on their different trajectories of, for instance, eating behaviors or weight (13). These approaches can examine trajectories of a single variable, or can consider trajectories based on multiple trajectory variables (14). For example, group-based trajectory modeling allows for analyses of trajectories of change across multiple variables to be considered when grouping participants (14, 15). This exploratory approach is primarily interested in understanding differences between individuals or subgroups in their eating behaviors and how they change over time. It can be useful for answering questions about group differences in the development of eating behaviors over infancy/childhood and can provide insights into the possible underlying mechanisms that can be tested in follow-up research.

A small number of studies from the United Kingdom and the United States have used person-centered approaches to identify appetitive phenotypes in childhood (1, 16–19), with some (1, 13, 18, 20, 21) examining appetitive phenotype trajectories or changes over time. In cross-sectional research, Boutelle et al. (13) identified three phenotypes from a combination of behavioral and self-report measures on a sample of 8- to 12-year-old North American children who were seeking treatment for overweight or obesity. Clairman et al. (22) also reported on three distinct phenotype groups in using multiple measures of appetite in overweight children/adolescents in Canada. Galloway et al. (20) reported three profiles of fussy eating based on scores from five subscales from the Children's Eating Behaviour Questionnaire (CEBQ). These studies have demonstrated how person-centered analyses of cross-sectional data can use multiple measures of appetitive behavior to create phenotypes, with these phenotypes highlighting individual subgroup differences in possible mechanisms in appetite self-regulation.

Further insights into appetite self-regulation and its development can be gained when data are longitudinal, and attention is directed to trajectories. Several studies have reported trajectories for individual appetitive traits: Fernandez et al. (21) identified three fussy eating phenotype trajectories in children from 4 to 9 years of age from low-income families in the United States. Also in the United States, Boutelle et al. (13) measured multiple appetitive traits at four time points in children (mean age 10 years) with overweight and obesity, and calculated trajectories for each of the traits. In the only published longitudinal study of appetite phenotypes beginning in infancy that we were aware of, Herle et al. (1) used latent class growth analysis to identify latent classes of trajectories of children's eating behaviors based on parents' reports of their concerns about their child's eating at six time points from age 15 months to 9 years. This study, using data collected in the Avon Longitudinal Study of Parents and Children (UK), found four classes of children for overeating and six each for undereating and fussy eating. In addition to the identification of latent classes, these studies also frequently investigated covariates or predictors and associations between the latent classes and outcome variables such as the BMI.

These studies have demonstrated that person-centered analyses of children's appetitive behaviors can provide additional insights into the development of children's eating behaviors beyond those identified with variable-centered approaches. Notably, person-centered approaches to the investigation of children's eating have the potential to contribute insights into individual differences, including individual differences in development. In the present research, we extend this approach in three ways: (1) the research is longitudinal rather than cross-sectional, thereby enabling the investigation of trajectories, (2) the analysis uses a multi-trajectory approach rather than an investigation of changes in individual variables, and (3) it focuses on infancy. This approach can provide insights into typologies of children's eating and appetite, including insights into the possible mechanisms.

The multi-trajectory approach used in the present study has been outlined by Nagin et al. (14). In this approach, the phenotypes are calculated from the trajectories of multiple variables. It conceptualizes phenotypes in terms of patterns of developmental pathways rather than patterns of measures taken cross-sectionally. However, although it is recognized that children's eating behaviors emerge early in life, to our knowledge, there are no studies describing appetitive phenotype trajectories using multiple indicators of appetite or eating in infants. Identifying appetitive phenotype trajectories using multiple, distinct trajectory variables in infancy contributes to knowledge on the origins of, and influences on, appetite self-regulation. That is, through understanding the features of the unique subgroups of eating, how they change in infancy and associate with covariates and outcomes, we are better able to understand the mechanisms and processes influencing the development of appetite self-regulation. It also helps to identify children on pathways likely to put them at greater risk of overweight or poor diet quality.

To better understand such trajectories and influences upon them, a biopsychosocial approach (23), which emphasizes understanding the development from birth or infancy as well as describing the processes and mechanisms that shape diet and weight over time, beginning with the biological characteristics of children can be useful. According to this model, through bidirectional and transactional processes, the impact of any emergent appetitive traits in infancy can be additive over time. In this way, it can help to explain developmental trajectories and identify opportunities for influencing such trajectories should they be considered problematic. This model informs the design and analysis of the current analysis where it is used to inform the examination of appetitive phenotype trajectories in infancy, the characteristics of infants (e.g., biological gender, birth weight) that are associated with different infant appetitive phenotype trajectories, and the environmental factors that influence those trajectories (23).

Considering the role of the environmental influences outlined in the biopsychosocial model, parent feeding practices have been identified as an important environmental factor related to children's appetitive traits (9, 24–26). Parent feeding practices and infant/child appetitive traits influence each other in contemporaneous, bidirectional, and transactional ways (27). Parent feeding practices are also influenced by parent cognitions such as attributions for the infant's behavior, perceptions of child/infant's weight as being too high or too low, or their own dieting and weight control cognitions (28, 29). Galloway et al. (20) linked parent feeding practices with child appetite phenotypes to show that parental pressure to eat was greater for children with a “picky eater” phenotype and lower for children with a “joyful” eating phenotype. Similarly, Fernandez et al. (21) observed differences in parent feeding between their groups of fussy eating phenotype trajectories. However, presently, the prevailing approach has been to associate such factors with individual appetitive traits rather than with infant appetitive phenotypes or phenotype trajectories. The role of parent feeding practices in appetitive phenotype trajectories of infants is therefore unclear and requires further exploration.

The present research, therefore, seeks to understand the early emergence of appetitive trait phenotypes in infants in the first year of life. The main aims were to (1) identify possible infant appetite phenotype trajectories, (2) examine relationships between infant appetite phenotype trajectories and infant/parent factors including infant weight and parent feeding practices.

## Materials and Methods

This was a longitudinal cohort study of infants and their parents recruited through an early parenting support service in Australia. Participants were asked to complete three surveys (time 1, time 2, and time 3), ~3 months apart, beginning when their infant was aged <6 months.

### Recruitment

Parents were recruited *via* Tresillian Family Care Centers, an early parenting support service (https://www.tresillian.org.au/) in New South Wales, Australia. At the Tresillian centers, flyers and posters were displayed around the buildings and nurses handed them to parents. Interested parents were then provided a Plain Language Statement and were given a hard copy of the questionnaire if they chose to participate. Parents returned the completed questionnaire to a sealed box at their Tresillian Center, which was subsequently collected by research staff. Parents were also recruited *via* advertisements on the Tresillian Facebook group. With this method, if parents responded to the advertisement they were linked to an electronic version of the Plain Language Statement and survey (which was hosted on SurveyGizmo). All participants who indicated a willingness at baseline to be contacted for follow-up and provided their email address were emailed an invitation to complete the subsequent survey 3 months after each survey completion, with reminders sent 1 week after the initial invitation. Surveys were hosted on SurveyGizmo. Eligibility criteria were: parent of an infant <6 months of age, parent aged 18 years, and able to read and write in English. Participants were excluded from analysis if their infant was >6 months of age at baseline, born at <35 weeks gestation, <2,500 g birthweight, living outside Australia, or had a health condition that affected feeding. Participants were offered the opportunity to enter into a draw to win one of two iPads. The University of Technology Sydney Human Research Ethics Committee (REF NO. 2015000528) and the Sydney Local Health District Human Research Ethics Committee (Protocol No X15-0233) granted ethical approval for the study.

### Questionnaire

#### Socio-Demographic Characteristics and Potential Confounders

The self-reported questionnaire included demographic variables: infant age and gender, parity, parent age and gender, parent relationship with child, parent level of feeding responsibility, infant feeding mode (breastfed, formula-fed, mixed-fed), and parent education level and country of birth. Potential confounders were infant feeding mode and several demographic characteristics: parent's age, parent's country of birth (Australia/other), parent's education (university/no university; categories collapsed from no formal qualification, finished year 12, post-school certificate, and university degree to ensure enough participants in each category), infant's gender (male/female), infant's age at baseline, and if ever formula-fed (yes/no).

#### Infant Appetitive Traits

Appetitive traits were measured with the BEBQ (2). The BEBQ consists of 18 items across four subscales (food responsiveness, enjoyment of food, slowness in eating, satiety responsiveness) and one single-item subscale (general appetite). The responses were recorded on Likert scales, ranging from 1 to 5 (never to always). After reversal of appropriate items, the item scores of each subscale were averaged to obtain a continuous score for each eating behavior. The BEBQ was originally designed to be a retrospective measure. However, in the present study, parents were asked about their infant's current behaviors. As such, wording of BEBQ items was changed to present tense (e.g., “my baby loved milk” was changed to “my baby loves milk”). The tool has shown good internal reliability (Cronbach's alpha coefficient of 0.62–0.77 at T1, 0.57–0.73 at T2, and 0.63–0.74).

#### Infant's Weight and Length

In each survey, parents reported their infant's most recent weight, length, and date of measurement from the infant's health record. Infant weight and length measurements recorded in the health record are taken at regular health check-ups with a health professional (e.g., nurses, general practitioners) using appropriate equipment. At the baseline survey, parents also reported their infant's birth weight and length recorded in their infant's health record (plus any measurements taken at a Tresillian center) and the date at which they were taken. To calculate the BMI *z*-scores, the World Health Organization's age- and gender-specific growth charts were used (30). BMI *z*-scores were used as a continuous variable.

#### Parent Cognitions and Characteristics

Parent perceptions of their infant's weight (underweight, about right, overweight), perception of their baby as being easier or more difficult than others, and parent self-reported height and weight (BMI) were considered as parent cognitions and characteristics.

#### Parent Feeding Practices

Parent feeding practices were measured with the Feeding Practices and Structure Questionnaire (FPSQ) for infants and toddlers (31). The FPSQ is theoretically grounded in the concept of authoritative feeding measuring both a parent's responsiveness to their child and provision of structure around mealtimes. The FPSQ infant and toddler version can either be used with parents who currently milk-feed their child (18 items) or solid-feed their child (21 items). At times 1 and 2 the milk feeding version was administered and at time 3 the solid feeding version was offered to parents who were predominantly solid-feeding (3+ meals or snacks per day) and the milk version to parents who were feeding solid foods <3 times per day. Four subscales of feeding practices were assessed: feeding on demand *vs*. feeding routine (e.g., “I let my baby decide when she/he would like to have a feed,” higher scores indicated more feeding on demand), using food to calm (e.g., “I feed my baby to make sure that she/he does not get unsettled or cry”), persuasive feeding (e.g., “If my baby indicates she/he is not hungry, I try to get him to feed anyway”), and parent-led feeding (e.g., “I feed my baby for a set time”). Responses were recorded on Likert scales, ranging from 1 to 5 (never to always). After reversal of appropriate items, the item scores of each subscale were averaged to obtain a continuous score for each feeding practice. This measurement tool was developed and validated in the current sample with good psychometric indicators, it showed good internal reliability with all Cronbach's alphas being above 0.7 (31).

### Statistical Analysis

The statistical analyses were conducted using Stata 16 (Stata Corp., College Station, TX, United States). Group-based trajectory modeling was utilized to identify the appetitive phenotype trajectories using continuous scores of the five scales from the BEBQ (satiety responsiveness, slowness in eating, food responsiveness, general appetite, and enjoyment of food) across the three times. The best-fitting model was determined based on the statistical model fit criteria, the class size, and the interpretation of the classes. The statistical criteria examined were the Bayesian Information Criteria (BIC), Consistent Akaike's Information Criteria (CAIC), Approximate Weight of Evidence Criterion (AWE), and the log-likelihood (32). The model that minimized the value of the BIC, CAIC, AWE, and the log-likelihood was determined to be the best fit statistically (32). The optimal number of classes was identified by analyzing 1-class through to 4-class models, with several polynomial types (linear, quadratic, and cubic) (32). The class sizes should be of sufficient size to examine the differences between the trajectory groups while class interpretation was based on looking at the average characteristics for the different variables included in the classes. Missing values were assumed to be missing completely at random, which was confirmed by checking each variable with the complete case.

Next, the mean and standard deviation or number and percentage of infant and parent characteristics, parent feeding practice, and cognitions were calculated for all participants, as well as for each appetitive phenotype. Chi-square, Fisher's exact test, and linear regression were used to test for associations between characteristics of infants and parents, parent feeding practice, and cognitions and Phenotypes.

Multilevel modeling was used to examine change in parent feeding practices and child BMI *z*-score over time by Phenotype. Participants had to have child BMI *z*-scores or parent feeding practices measured at least two of the times to be included in the analysis since multilevel modeling permits subjects with missing outcome data at some of the time points (33). Model 1 included outcome measures (i.e., child BMI *z*-score and parent feeding practices) by time. Model 2 included outcomes measures by time, appetitive phenotype trajectory groups, and time ^*^ appetitive phenotype trajectory groups. While Model 3 included Model 2 plus potential confounders (parent's age, parent's country of birth, parent's education, child's gender, child's age, and BMI *z*-score at birth).

A multilevel linear regression model was used to model the outcome measures (child BMI *z*-score and parent feeding practices) by time (model 1) to determine if child BMI *z*-score and parent feeding practices changed over time for all participants. For child BMI *z*-score, this examination was from birth to time 3, for parent feeding practices this examination was from time 1 to time 3. Likelihood ratio tests were used to determine if the model fit improved by including a random slope in addition to a random intercept. It was determined that the inclusion of a random slope was better for assessing the change in BMI *z*-score over time. However, including a random intercept only was better for assessing the change in parent feeding practices over time. To assess whether change in child BMI *z*-score and parent feeding practices over time differed depending on the Phenotype, an interaction between Phenotype and time was included in the model, in addition to the main effect of time and Phenotype (model 2). Potential confounding variables were included next (model 3). Phenotype 1 was considered as the reference category in models 2 and 3.

To interpret the interaction effects, a *post-hoc* analysis was conducted (predictive margins test) (34) to estimate the change in child BMI *z*-score and parent feeding practices over time for each of the three multi-trajectory appetitive phenotype groups.

## Results

### Sample Characteristics

Table 1 shows the characteristics at time 1 of the total sample and for each appetitive phenotype trajectory group. In total, 445 provided some data; 65 participants were excluded, leaving 380 participants at baseline, 178 at time 2, and 154 at time 3 (Table 1). At time 1, just over half of the participating infants were men (54.8%) and all but one (father) was the infant's mother. The mean age of the children at time 1 was 98 (range: 5–183) days, at time 2 it was 198 (98–294), and at time 3 it was 303 (193–401) days. The mean BMI *z*-score for all children at birth was −0.08 (SD = 1.17), at time 1 it was −0.28 (SD = 1.31), at time 2 it was 0.27 (SD=1.35), and the mean BMI *z*-score at time 3 was 0.49 (SD = 1.37). Of the 380 children who participated at baseline, 335 (88%) had BMI *z*-score reported at least twice, while 182 (48%) had parent feeding practices reported at least twice, to enable inclusion in the longitudinal analysis. Other reasons for exclusion were birthweight <2,500 g, living outside of Australia, gestation <35 weeks, if the infant was older than 6 months at baseline, or had a health condition affecting feeding.

**Table 1:** Characteristics of the participants at time 1 and by Phenotype (*n* = 380).

**Variable**	**All participants**	**Phenotype 1** **(*n* = 81, 21.32%)**	**Phenotype 2** **(*n* = 192, 50.53%)**	**Phenotype 3** **(*n* = 107, 28.16%)**	***P*** **-value**
***n*** **(%) or mean (SD)**					
Infant characteristics					
BMI *z*-score at birth	−0.08 (1.17)	−0.13 (1.03)	−0.17 (1.27)	0.11 (1.07)	0.128
Child age (days)	98.09 (47.39)	101.96 (43.63)	98.86 (49.00)	93.78 (47.27)	0.192
Child gender					0.139
Male	206 (54.79)	36 (45.00)	108 (57.14)	62 (57.94)	
Female	170 (45.21)	44 (55.00)	81 (42.63)	45 (42.45)	
Ever formula fed					0.016^*^
Yes	138 (36.32)	40 (49.38)	66 (34.55)	32 (29.63)	
No	242 (63.68)	41 (50.62)	125 (65.45)	76 (70.37)	
**Parent characteristics**					
Parent education					0.713
University	219 (59.67)	49 (63.64)	111 (59.04)	59 (58.42)	
No university	148 (40.33)	28 (36.36)	77 (40.96)	43 (42.16)	
Parent age (years)					0.689
29 and under	149 (39.95)	27 (33.75)	77 (40.96)	45 (42.86)	
30–34	146 (39.14)	36 (45.00)	73 (38.83)	37 (35.24)	
35 and over	78 (20.91)	17 (21.25)	38 (20.21)	23 (21.90)	
Country of birth					0.148
Australia	318 (85.71)	73 (92.41)	157 (83.96)	88 (83.81)	
Other	53 (14.29)	6 (7.59)	30 (16.04)	17 (16.19)	
Parent BMI	27.75 (6.09)	27.63 (6.39)	27.93 (6.01)	27.52 (6.04)	0.901

* *p < 0.05. Bold values indicate statistical significance*.

### Appetitive Phenotype Trajectories

**Figure 1:**
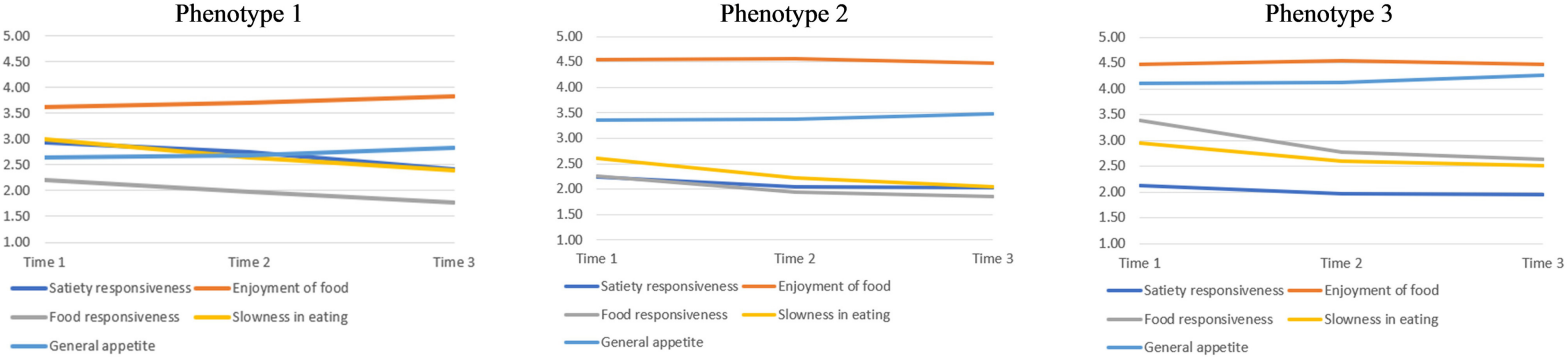
The multi-trajectory appetitive phenotype groups (Phenotype 1, Phenotype 2, and Phenotype 3, respectively) and their Baby Eating Behavior Questionnaire (BEBQ) subscale trajectories.

The three-group solution of multi-trajectory appetitive phenotype groups, herein called “Phenotypes,” was chosen based on class size, interpretation, and statistical model fit (lowest values for LL). Figure 1 shows the mean for each appetitive trait trajectory within each phenotype at each time point. The majority (*n* = 192; 51%) of infants were in Phenotype 2 (see description below), with Phenotype 3 representing 28% (*n* = 107) of the sample, and the remaining 21% (*n* = 81) of infants in Phenotype 1.

The appetitive traits at each time point for each appetitive phenotype trajectory group are shown in Table 2. At baseline, Phenotype 1 had the lowest mean score over time for enjoyment of food, food responsiveness, and general appetite while having the highest score for satiety responsiveness and slowness in eating, suggesting a more “food avoidant” phenotype. In contrast, Phenotype 3 and Phenotype 2 both had high scores for the enjoyment of food. General appetite was particularly high in Phenotype 3, coupled with the highest scores for food responsiveness and lowest scores for satiety responsiveness. Phenotype 2 represented a relatively balanced phenotype at baseline, scoring relatively high on “food approach” appetitive traits (particularly enjoyment of food), but somewhere in between the two other phenotypes. In terms of changes over time, mean score differences in the appetitive traits scores across the three multi-trajectory phenotype groups were similar at time 2 and time 3, yet there were differences in the trajectories of the BEBQ subscales: Phenotype 1 showed increasing enjoyment of food and general appetite, along with decreasing slowness in eating, food responsiveness, and satiety responsiveness. Phenotype 2 showed stable enjoyment of food, increasing general appetite, and decreasing slowness in eating, satiety responsiveness, and food responsiveness. Phenotype 3 also showed decreasing slowness in eating and food responsiveness, along with increasing general appetite, while enjoyment of food remained relatively stable at high levels and satiety responsiveness relatively stable at low levels.

**Table 2:** Appetitive traits (Baby Eating Behavior Questionnaire) and feeding practices scores for each time point according to Phenotypes.

**Variable (BEBQ subscales)**	**All participants** **(*n* = 380)**	**Phenotype 1** **(*n* = 81, 21.32%)**	**Phenotype 2** **(*n* = 192, 50.53%)**	**Phenotype 3** **(*n* = 107, 28.16%)**
**Mean (SD)**				
Time 1 (mean age in days = 80)				
Satiety responsiveness	2.35 (0.79)	2.94 (0.93)	2.23 (0.66)	2.12 (0.66)
Slowness in eating	2.78 (0.84)	3.00 (0.90)	2.61 (0.77)	2.95 (0.83)
Food responsiveness	2.57 (0.74)	2.20 (0.60)	2.26 (0.49)	3.38 (0.58)
General appetite	3.42 (0.93)	2.63 (0.80)	3.36 (0.73)	4.12 (0.81)
Enjoyment of food	4.33 (0.58)	3.62 (0.56)	4.55 (0.43)	4.47 (0.39)
Feeding on demand	2.02 (0.84)	2.34 (0.09)	2.01 (0.07)	1.81 (0.07)
Food to calm	2.54 (0.83)	2.49 (0.08)	2.38 (0.06)	2.84 (0.08)
Parent-led feeding	1.62 (0.69)	1.83 (0.07)	1.58 (0.05)	1.55 (0.07)
Persuasive feeding	1.94 (0.75)	2.22 (0.09)	1.75 (0.05)	2.08 (0.08)
Time 2 (mean age in days = 171)				
Satiety responsiveness	2.17 (0.74)	2.75 (0.80)	2.04 (0.68)	1.96 (0.58)
Slowness in eating	2.42 (0.73)	2.65 (0.78)	2.22 (0.65)	2.60 (0.74)
Food responsiveness	2.18 (0.68)	1.97 (0.49)	1.94 (0.61)	2.77 (0.57)
General appetite	3.45 (0.86)	2.69 (0.75)	3.37 (0.71)	4.12 (0.63)
Enjoyment of food	4.37 (0.59)	3.70 (0.48)	4.56 (0.43)	4.55 (0.53)
Feeding on demand	2.22 (0.83)	2.47 (0.13)	2.80 (0.09)	1.92 (0.10)
Food to calm	2.41 (0.85)	2.38 (0.13)	2.22 (0.08)	2.76 (0.12)
Parent-led feeding	1.78 (0.67)	1.97 (0.11)	1.78 (0.08)	1.62 (0.08)
Persuasive feeding	1.84 (0.64)	2.10 (0.12)	1.66 (0.06)	1.94 (0.09)
Time 3 (mean age in days = 271)				
Satiety responsiveness	2.06 (0.76)	2.41 (1.06)	2.03 (0.70)	1.93 (0.60)
Slowness in eating	2.25 (0.73)	2.40 (0.70)	2.04 (0.64)	2.52 (0.78)
Food responsiveness	2.08 (0.68)	1.76 (0.55)	1.85 (0.55)	2.63 (0.62)
General appetite	3.62 (0.99)	2.84 (0.85)	3.48 (0.88)	4.26 (0.85)
Enjoyment of food	4.37 (0.50)	3.83 (0.56)	4.48 (0.39)	4.49 (0.45)
Feeding on demand	3.49 (0.70)	3.58 (0.15)	3.57 (0.09)	2.34 (0.10)
Food to calm	1.83 (0.54)	1.81 (0.13)	1.83 (0.07)	1.86 (0.08)
Parent-led feeding	1.94 (0.68)	1.99 (0.12)	1.96 (0.07)	1.89 (0.10)
Persuasive feeding	2.44 (0.65)	2.55 (0.13)	2.40 (0.08)	2.44 (0.10)

*Subscales of the Baby Eating Behavior Questionnaire (BEBQ) (2) and Feeding Practices and Structure Questionnaire (FPSQ) for infants and toddlers, both with a possible range from 1 to 5 (31)*.

### Characteristics of Infants and Parents According to Phenotypes

As shown in Table 1, compared to infants in Phenotypes 2 or 3, infants in Phenotype 1 were more likely to be formula fed (*p* = 0.016). There was no evidence of differences in the other measured infant or parent characteristics according to the multi-trajectory appetitive phenotype group.

### Associations Between Parent Feeding Practices and Phenotypes

**Figure 2:**
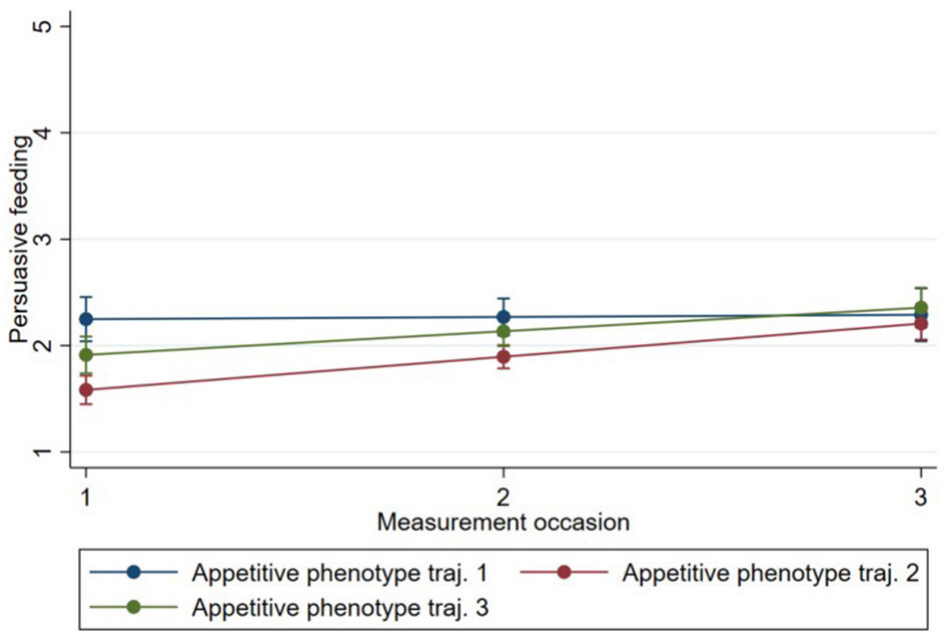
Change in “persuasive feeding” over time by Phenotype based on estimates from fully adjusted multilevel model of the association between Phenotype and persuasive feeding.

**Figure 3:**
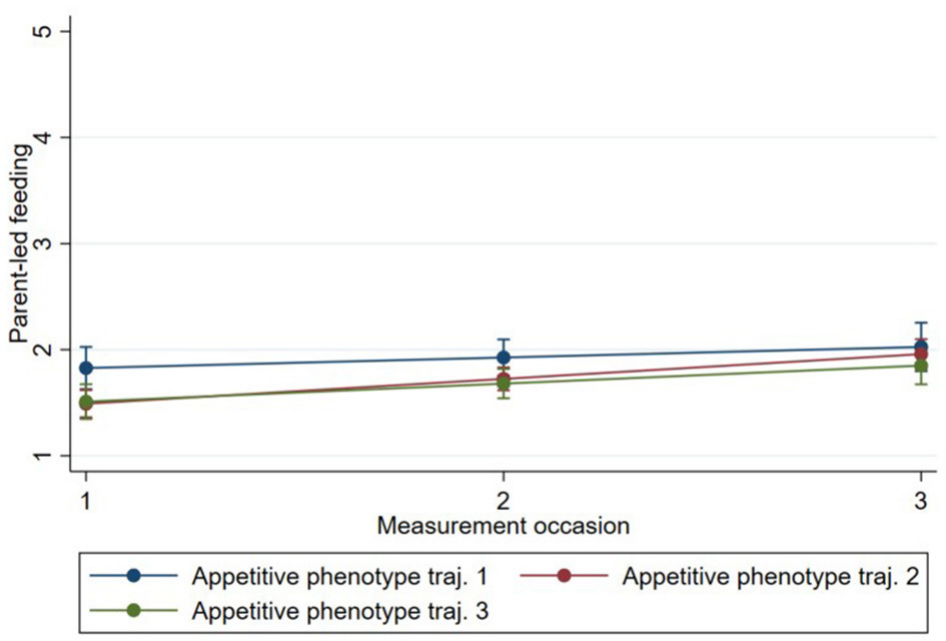
Change in “parent-led feeding” over time by Phenotype based on estimates from fully adjusted multilevel model of the association between Phenotype and “parent-led feeding”.

**Figure 4:**
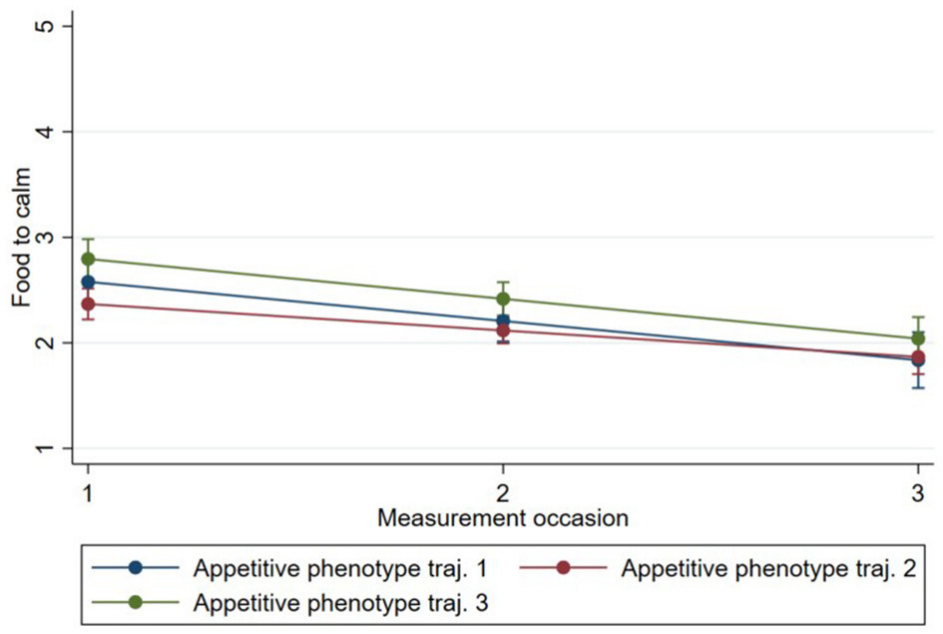
Change in “using food to calm” over time by Phenotype based on estimates from fully adjusted multilevel model of the association between Phenotype and “using food to calm”.

**Figure 5:**
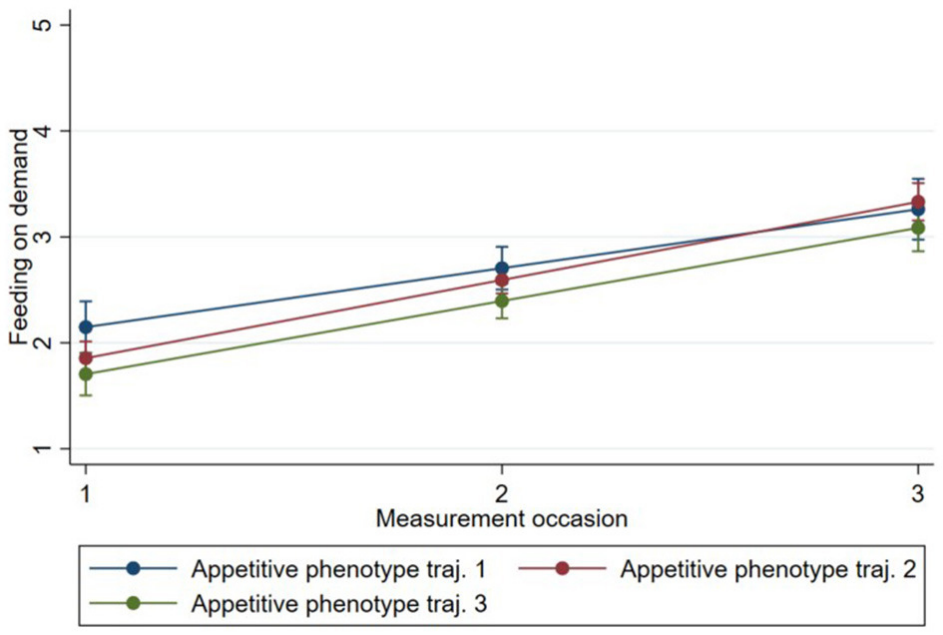
Change in “feeding on demand” over time by Phenotype based on estimates from fully adjusted multilevel model of the association between Phenotype and “feeding on demand”.

Model 1 showed that “feeding on demand” [0.69, 95% CI (0.62, 0.77)], “persuasive feeding” [0.22, 95% CI (0.16, 0.29)], and “parent-led feeding” [0.19, 95% CI (0.13, 0.24)] increased on average between time 1 and time 3 for all phenotypes, while “using food to calm” [−0.31, 95% CI (−0.37,−0.25)] decreased between time 1 and time 3 for all phenotypes (Tables 3–6). The estimates of subsequent models (2 and 3) from the multilevel models are also presented in Tables 3–6. There was evidence that change in “persuasive feeding” differed according to infant phenotypes (Figure 2). Findings from the *post-hoc* analysis showed that Phenotype 2 had the greatest increase in “persuasive feeding” over time [0.30; 95% CI (0.12, −0.47)], while Phenotype 3 [0.21; 95% CI (0.01, −0.39)] and Phenotype 1 [0.02; 95% CI (−0.13, 0.17)] showed less increase over time. There was no evidence that the change in “feeding on demand,” “parent-led feeding,” or “using food to calm” over time differed according to the phenotypes (Figures 3–5).

**Table 3:** Multilevel models of associations between Phenotypes and “parent-led feeding” over three times (*n* = 182).

**Fixed effects**	**Model 1**			**Model 2**			**Model 3** ^a^		
	**Coefficient**	**95% CI**	***P*** **-value**	**Coefficient**	**95% CI**	***P*** **-value**	**Coefficient**	**95% CI**	***P*** **-value**
Time	0.19	0.13, 0.24	<0.001^***^	0.10	−0.02, 0.23	0.112	0.10	−0.03, 0.23	0.133
Appetitive phenotype									
Phenoty pe 1				–	–	–	–	–	–
Phenotype 2				−0.30	−0.54, −0.07	0.011^*^	−0.34	−0.58, −0.10	0.005^**^
Phenotype 3				−0.33	−0.59, −0.08	0.010^*^	−0.31	−0.57, −0.05	0.018^*^
Appetitive phenotype × time									
Phenotype 1 × time				–	–	–	–	–	–
Phenotype 2 × time				0.13	−0.02, 0.28	0.096	0.14	−0.02, 0.29	0.081
Phenotype 3 × time				0.07	−0.09, 0.23	0.398	0.07	−0.09, 0.23	0.386

* *p < 0.05,*

** *p < 0.01,*

*** *p < 0.001. Bold values indicate statistical significance.*

a *Model 3 adjusted for parent's age, parent's country of birth, parent's education, child's gender, child's age, and BMI z-score at birth*.

**Table 4:** Multilevel models of associations between Phenotypes and “persuasive feeding” over three times (*n* = 182).

**Fixed effects**	**Model 1**			**Model 2**			**Model 3^a^**		
	**Coefficient**	**95% CI**	***P*** **-value**	**Coefficient**	**95% CI**	***P*** **-value**	**Coefficient**	**95% CI**	***P*** **-value**
Time	0.22	0.16, 0.29	<0.001^***^	0.03	−0.12, 0.18	0.677	0.02	−0.13, 0.17	0.789
Appetitive phenotype									
Phenotype 1				–	–	–	–	–	–
Phenotype 2				−0.64	−0.88, −0.41	<0.001^***^	−0.67	−0.92, −0.42	<0.001^***^
Phenotype 3				−0.31	−0.57, −0.05	0.019^*^	−0.34	−0.61, 0.06	0.015^*^
Appetitive phenotype × time									
Phenotype 1 × time				–	–	–	–	–	–
Phenotype 2 × time				0.29	0.12, 0.46	0.001^**^	0.30	0.12, 0.47	0.001^**^
Phenotype 3 × time				0.17	−0.01, 0.35	0.071	0.21	0.01, 0.39	0.030^*^

* *p < 0.05,*

** *p < 0.01,*

*** *p < 0.001. Bold values indicate statistical significance.*

a *Model 3 adjusted for parent's age, parent's country of birth, parent's education, child's gender, child's age, and BMI z-score at birth*.

**Table 5:** Multilevel models of associations between Phenotypes and “feeding on demand” over three times (*n* = 182).

**Fixed effects**	**Model 1**			**Model 2**			**Model 3** ^a^		
	**Coefficient**	**95% CI**	***P*** **-value**	**Coefficient**	**95% CI**	***P*** **-value**	**Coefficient**	**95% CI**	***P*** **-value**
Time	0.69	0.62, 0.77	<0.001^***^	0.55	0.38, 0.72	0.001^**^	0.56	0.38, 0.73	<0.001^***^
Appetitive phenotype									
Phenotype 1				–	–	–	–	–	–
Phenotype 2				−0.37	−0.66, −0.08	0.011^*^	−0.32	−0.60, −0.03	0.032^*^
Phenotype 3				−0.53	−0.84, −0.22	0.001^*^	−0.44	−0.75, −0.12	**0.007** ^**^
Appetitive phenotype × time									
Phenotype 1 × time				–	–	–	–	–	–
Phenotype 2 × time				0.20	−0.00, 0.40	0.050	0.19	−0.01, 0.40	0.065
Phenotype 3 × time				0.15	−0.06, 0.36	0.158	0.14	−0.08, 0.36	0.200

* *p < 0.05,*

** *p < 0.01,*

*** *p < 0.001. Bold values indicate statistical significance.*

a *Model 3 adjusted for parent's age, parent's country of birth, parent's education, child's gender, child's age, and BMI z-score at birth*.

**Table 6:** Multilevel models of associations between Phenotypes and “using food to calm” over three times (*n* = 182).

**Fixed effects**	**Model 1**			**Model 2**			**Model 3** ^a^		
	**Coefficient**	**95% CI**	***P*** **-value**	**Coefficient**	**95% CI**	***P*** **-value**	**Coefficient**	**95% CI**	***P*** **-value**
Time	−0.31	−0.37, −0.25	<0.001^***^	−0.36	−0.51, −0.21	<0.001^***^	−0.37	−0.52, −0.22	<0.001^***^
Appetitive phenotype									
Phenotype 1				–	–	–	–	–	–
Phenotype 2				−0.16	−0.42, 0.11	0.249	−0.20	−0.47, 0.07	0.153
Phenotype 3				0.28	−0.02, 0.56	0.064	0.21	−0.09, 0.51	0.172
Appetitive phenotype × time									
Phenotype 1 × time				–	–	–	–	–	–
Phenotype 2 × time				0.12	−0.06, 0.29	0.194	0.11	−0.07, 0.29	0.212
Phenotype 3 × time				−0.02	−0.21, 0.16	0.806	−0.00	−0.19, 0.19	0.974

*** *p < 0.001. Bold values indicate statistical significance.*

a *Model 3 adjusted for parent's age, parent's country of birth, parent's education, child's gender, child's age, and BMI z-score at birth*.

### Associations Between Parent Cognitions and Infant Phenotypes

Parents reported that compared to the other appetitive phenotypes, at time 1 infants in Phenotype 1 were more likely (*p* = 0.001) to be seen as “more difficult than average,” with a similar finding at time 2 (*p* = 0.018) and 3 (*p* = 0.017) (Table 7). The majority of parents indicated that their infant's weight was “about right” at each time, with statistical significance noted between the phenotypes at time 1 (*p* = 0.012), but not at time 2 and time 3.

**Table 7:** Parent cognitions according to Phenotypes.

	**All participants** **(*n* = 380)**	**Phenotype 1** **(*n* = 81, 21.32%)**	**Phenotype 2** **(*n* = 192, 50.53%)**	**Phenotype 3** **(*n* = 107, 28.16%)**	***P*** **-value**
***n*** **(%) or mean (SD)**					
Parent cognitions					
Perception of baby compared to others at time 1					0.001^**^
Easier than average	162 (43.67)	23 (29.87)	93 (49.47)	46 (43.40)	
Average	139 (37.47)	28 (36.36)	72 (38.30)	39 (36.79)	
More difficult than average	70 (18.87)	26 (33.77)	23 (12.23)	21 (19.81)	
Perception of baby compared to others at time 2					0.018^*^
Easier than average	89 (52.05)	13 (35.14)	55 (63.95)	21 (43.75)	
Average	65 (38.01)	18 (48.65)	24 (27.91)	23 (47.92)	
More difficult than average	17 (9.94)	6 (16.22)	7 (8.14)	4 (8.33)	
Perception of baby compared to others at time 3					0.017^*^
Easier than average	80 (54.42)	7 (28)	45 (60.81)	28 (58.33)	
Average	45 (30.61)	9 (36)	21 (28.38)	15 (31.25)	
More difficult than average	22 (14.97)	9 (36)	8 (10.81)	5 (10.42)	
Perception of baby's weight at time 1					0.012^*^
Underweight	19 (5.12)	10 (12.99)	4 (2.13)	5 (4.72)	
About right	336 (90.57)	65 (84.42)	176 (93.62)	95 (89.62)	
Overweight	16 (4.31)	2 (2.6)	8 (4.26)	6 (5.66)	
Perception of baby's weight at time 2					0.649
Underweight	6 (3.51)	2 (5.41)	4 (4.65)	0 (0.00)	
About right	155 (90.64)	33(89.19)	77 (89.53)	45 (93.75)	
Overweight	10 (5.85)	2 (5.41)	5 (5.81)	3 (6.25)	
Perception of baby's weight at time 3					0.278
Underweight	8 (5.44)	3 (12)	4 (5.41)	1 (2.08)	
About right	130 (88.44)	21 (84)	67 (90.54)	42 (87.50)	
Overweight	9 (6.12)	1 (4)	3 (4.05)	5 (10.42)	

* *p < 0.05,*

** *p < 0.01. Bold values indicate statistical significance*.

### Associations Between Infant BMI *z*-Score and Infant Phenotypes

**Figure 6:**
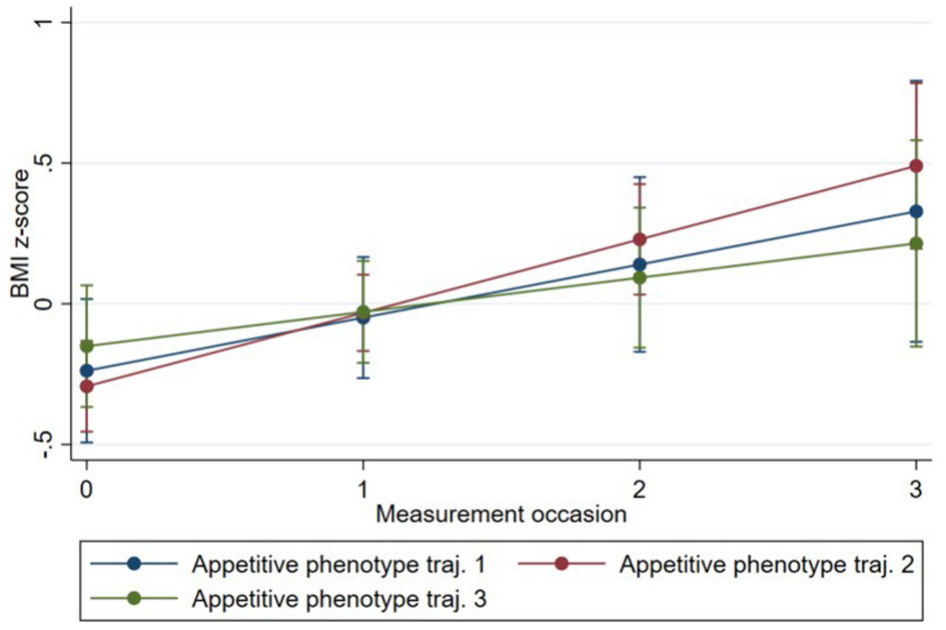
Change in infant BMI *z*-score over time by Phenotype based on estimates from fully adjusted multilevel model of the association between Phenotype and infant BMI *z*-score.

Model 1 showed that BMI *z*-scores increased on average between birth and time 3 [0.21, 95% CI (0.12, 0.29)]. The estimates of subsequent models (2 and 3) from the multilevel models are presented in Table 8. There was no evidence that BMI *z*-score change over time differed depending on the phenotype. However, an inspection of trends showed that children in Phenotype 3 had the highest BMI *z*-score at birth, yet findings from the *post-hoc* analysis showed that children in Phenotype 3 had the smallest incline of BMI *z*-score over time [−0.07; 95% CI (−0.31, 0.17)]. Phenotypes 1 and 2 had lower BMI *z*-score at birth, while Phenotype 2 had the highest BMI *z*-score at time 3. These findings are illustrated in Figure 6.

**Table 8:** Multilevel models of associations between Phenotypes and BMI *z*-score over four times (*n* = 335).

**Fixed effects**	**Model 1**			**Model 2**			**Model 3** ^a^		
	**Coefficient**	**95% CI**	***P*** **-value**	**Coefficient**	**95% CI**	***P*** **-value**	**Coefficient**	**95% CI**	***P*** **-value**
Time	0.21	0.12, 0.29	<0.001^***^	0.19	0.01, 0.37	0.041^*^	0.19	0.00, 0.37	0.046^*^
Appetitive phenotype									
Phenotype 1				–	–	–	–	–	–
Phenotype 2				−0.11	−0.41, 0.18	0.453	−0.06	−0.36, 0.24	0.687
Phenotype 3				0.05	−0.28, 0.38	0.771	0.09	−0.24, 0.43	0.589
Appetitive phenotype × time									
Phenotype 1 × time				–	–	–	–	–	–
Phenotype 2 × time				0.07	−0.15, 0.29	0.545	0.07	−0.15, 0.29	0.529
Phenotype 3 × time				−0.06	−0.29, 0.18	0.635	−0.07	−0.31, 0.17	0.560

* *p < 0.05,*

*** *p < 0.001. Bold values indicate statistical significance.*

a *Model 3 adjusted for parent's age, parent's country of birth, parent's education, child's gender, child's age, and formula feeding*.

## Discussion

This study described three empirically distinct appetitive phenotype trajectory groups among a group of Australian infants labeled as (Phenotype 1) food avoidant trending toward low food approach (Phenotype 2), balanced, and (Phenotype 3) high and continuing food approach. There was no evidence to support infant or parent demographic characteristics, nor infant BMI *z*-score differing according to the phenotypes. However, for the measured parent feeding practices, persuasive feeding changed over time according to phenotypes while for parent cognitions, perceptions of the infant's weight and how difficult or easy they were differed according to the phenotypes. The findings provide novel evidence suggesting that distinct infant appetite phenotype trajectories emerge early in life, and may partly have their origins in both infant characteristics and eating experiences, as well as being related to parent feeding practices and cognitions. The findings aid our understanding of when and how appetite self-regulation develops and highlights the need for a greater focus on person-centered approaches to understanding appetite self-regulation in infancy.

Previous research has shown that differences in approaches to eating (appetitive traits) emerge early in life (1–3, 35). However, this body of work has primarily examined mean scores across individual appetitive traits, and not examined profiles of infants based on appetitive traits, as an appetitive phenotype, nor examined appetite phenotype trajectories either with single or multiple indicators of appetite. Consequently, the heterogeneity in infant appetite profiles across the course of infancy has often been overlooked, and therefore our understanding of the developmental course of appetite self-regulation is hindered. Our findings provide new evidence that distinct phenotypes of appetitive trait trajectories emerge early in infancy. In the present study, Phenotype 2 (50.53% of infants) was considered “balanced” (normal), showing a relatively high general enjoyment of food and appetite with decreasing satiety responsiveness and food responsiveness over time, starting from a moderate level. Phenotype 1 (21.32% of infants) was relatively low in food enjoyment and appetite while being relatively higher in satiety responsiveness and slowness in eating, with food responsiveness decreasing over time, suggesting that these children have a food avoidance phenotype initially. However, trajectories of the BEBQ subscales in Phenotype 1 are suggestive of a shift toward greater (but still relatively low) food approach tendencies over time with general appetite, enjoyment of food increasing at the same time as slowness in eating and satiety responsiveness are decreasing. Phenotype 3 (28.16% of infants), in contrast, was relatively higher in the enjoyment of food and appetite initially, while being low in satiety responsiveness. This phenotype showed increasing levels of general appetite in combination with reduced levels of slowness in eating while maintaining high levels of enjoyment of food and low levels of satiety responsiveness. These early signs suggest that this appetitive phenotype trajectory group is likely to promote excess weight gain if the trajectories were to continue along the same path. It is also worth noting that this phenotype did not differ from the others in terms of parent demographics (e.g., education level, country of birth), nor child's biological gender highlighting the need to tailor personalized obesity prevention approaches to their unique appetitive trait profile and influences upon it, rather than demographic characteristics (23). Infancy (36) toddlerhood, and the preschool years are developmental periods when children rapidly learn about food and eating (37), and it would be valuable to explore whether these trends continue across later periods of development.

Phenotype 1, which appeared to have a food avoidant profile at baseline, could also be considered in obesity prevention efforts. The trajectories observed within this phenotype included increasing enjoyment of food and general appetite along with decreasing slowness in eating, emulating the trends seen in Phenotype 3 despite different mean levels. Although there are relatively fewer studies with infants than there are with children, a systematic analysis of the BEBQ identified prospective associations between variables measuring food responsiveness, enjoyment of food, general appetite, and higher adiposity; while satiety responsiveness and slowness in eating were prospectively associated with lower adiposity (4). It could therefore be expected that in the present study, Phenotype 1 would be associated with a lower BMI *z*-score, and Phenotype 3 with a higher BMI *z*-score. While there was no evidence that BMI *z*-scores differed dependent on phenotype over time in the present study, it is likely that any effects of the appetitive phenotypes on BMI would only be observed over longer timeframes due to the cumulative direct and indirect effects of the appetitive phenotype profiles on food intakes (27). It is also worth noting that infants in Phenotype 1 differed from the other phenotypes in other characteristics: parents perceived them as “more difficult than average” at both time 1 and time 2, more infants were perceived as being underweight at time 1, and they were more likely to have been formula-fed. It is possible that, broadly, these infants inherently have low interest in food and smaller appetites, along with being seen by their parents as having more difficult temperaments and being underweight early on. This broadly supports the ideas outlined in biopsychosocial models of children's eating and weight, whereby combinations of infant characteristics, parent perceptions, and parent feeding practices, like those observed here, can help explain trends in the development of children's appetitive traits, and over greater time periods where additive effects may be evident, may also influence weight (27).

The examination of feeding practices revealed that “feeding on demand,” “persuasive feeding,” and “parent-led feeding” increased over time, while “using food to calm” decreased over time for all three appetitive phenotype trajectory groups. However, of the four measured feeding practices, only the change in persuasive feeding was related to phenotype: Phenotype 2 had the greatest increase in persuasive feeding over time, while phenotypes 3, and 1 showed less increase over time. The “persuasive feeding” subscale represents non-responsive feeding practices that are likely to negatively impact the development of aspects of appetite self-regulation (31). Although it is speculative due to the short-term nature of the study, the upward trend in the BMI *z*-score observed in this group (Figure 6) may be at least partly attributed to the greater use of non-responsive parent feeding practices in this group. So, the findings suggest that while, in general, parent feeding practices change with children's development in many common ways, some differences can already be observed in the use of particular parent feeding practices according to infants' appetite phenotypes. This concurs somewhat with previous research showing that parental feeding practices are associated with appetitive phenotypes in children (20) and that parent feeding practices are both reactive to, and influence infant/child eating and weight (9, 10, 38). These findings highlight the need for future research over longer periods with age-appropriate repeated measurement of key constructs to identify how and why early parent feeding practices affect and are affected by infant appetitive phenotypes and can affect appetite infant self-regulation.

### Strengths and Weaknesses

Strengths include a sample that involved a balanced proportion of male and female infants, and the use of an age- and feeding-mode appropriate measure of parent feeding practices. However, the present study was limited in its reliance upon a single informant, and is therefore subject to common method bias (e.g., potentially inflating correlations between parent perceptions of how easy/difficult the infant is and their appetitive traits) (39–41). It was also reliant upon the parent-reported BEBQ, which is subject to several biases and limitations including limited data on validity, and parent recall and social desirability bias, and poor factorial validity (35) and it was originally developed for use retrospectively with younger, exclusively milk-fed infants (2). The reliance upon parent-reported infant height and weight is also a limitation, although the use of the infant health record for weight and length measurements as well as using concurrent (rather than retrospective) measures of the BEBQ would have tempered some of these limitations. In addition, there are probably interrelations between variables such as between parent perceptions and infant appetitive traits, and these could not be teased out with the current study. There was also a lag between the date of weight/length measurements and the date at which parents completed the survey at each time point (a mean of 18 days at T1, 24 days at T2, and 34 days at T3), which reduces the precision of that variable. A combination of behavioral and parent-reported parent measures of infant appetitive characteristics as well as utilizing data from multiple informants (e.g., from more than one caregiver) would have strengthened the present research.

The present study examined infants over ~6 months with parents enrolling in the study when their infants were aged <6 months of age. This meant that at each time point the age range of infants was wide, and this may have influenced the appearance of appetitive traits over time. It could be useful in future studies to limit each time point to a narrower age band. The present study was also relatively short in duration, while traversing the transition from milk to solid foods, which is a period of change and adjustment in parent feeding and infant eating. Feeding interactions change quickly with infants, and therefore we have captured a snapshot moment that might not be reflective of longer-term trends in infant eating and parent feeding interactions. To address this, studies of longer duration are needed to understand the different trajectories, how infants and parents within each of these trajectories change over time, and how they relate to a range of dietary and weight outcomes. The person-centered, group-based trajectory modeling approach allowed for distinct trajectories of infant eating behaviors to be examined. However, this approach is subject to limitations that affect the interpretability of findings. In particular, larger or different samples may result in different subgroups being identified, and so reproducibility of the current findings should be tested in other, larger samples. It should also be noted that missing data due to dropout was high. Multilevel models permit subjects with missing outcome data at some of the time points, so this helped reduce the risk of bias for the BMI *z*-score outcome. However, as we only had three time points for the feeding practices, the outcome risk of bias due to dropout may still be high. We also performed additional analyses to see if there was retention bias, with only parental education level being of concern: slightly more university-educated parents did not drop out of the survey (60% of the sample at T1 were university-educated while at T3 this was 70%), a common issue found in longitudinal studies relying on parents of young children. Future studies with larger sample sizes and lower dropout rates would be beneficial.

### Future Directions

Looking forward, identifying and understanding early predispositions toward overeating, food avoidance, or healthy eating as well as the factors that explain their development is important for understanding how and why appetite self-regulation develops. In general, children's appetite self-regulation declines from infancy across childhood, although large individual differences are evident (42). Prior research on eating phenotypes has mostly examined older children and has utilized variable-centered approaches, and so the early origins of appetitive phenotypes are largely unknown. In studying the emergence of appetitive phenotypes and their changes in infancy, new insights are gained when examining individual differences in appetite self-regulation and its possible early origins. This information is needed to advance the theory and conceptualization of appetite self-regulation and to inform early intervention to address such traits before the development of overweight (43). The present research has provided new evidence that early in infancy there are signs that infants already have particular typologies of eating, and that these may set in train patterns of eating and possibly later weight outcomes. The present study also identified that these appetitive phenotypes appear to have their origins in both infant (e.g., birth weight) and parent characteristics and behaviors (e.g., perception of the infant, feeding practices). To better explain the processes underlying the development of infant appetitive phenotypes, studies informed by biopsychosocial models of eating and weight are needed. The biological origins of children's appetite and temperament are important components of developmental pathways, along with the psychological and social contexts that interact with these biological characteristics. Future studies that are able to elucidate the complex changes in both infant appetitive characteristics along with the factors that influence their development are needed (44). Looking beyond cross-sectional differences in children's phenotypes at baseline to understand developmental patterns of change and the processes explaining these changes will provide greater insights into the origins of, and influences on eating behaviors. These approaches will also help improve the utility of interventions aiming to improve children's diets and prevent excess weight gain by allowing for better matching of intervention features with infants' or family's needs (5, 13). In addition, appetitive phenotypes will be composed of several factors affecting eating and appetite and so will be a function of the selected measures. Further work to identify the relevant components of appetitive phenotypes, including their interactions and synergistic effects, across biological, behavioral and psychological factors of eating and appetite, with attendant appropriate measurement, is important for understanding trajectories of eating and appetite (5, 43). To that end, the study of appetitive traits in infants would be advanced by the development of additional age-appropriate tools and measurements beyond that of the BEBQ, that are suitable for different samples and contexts.

## Conclusions

The present study identified three appetitive phenotype trajectory groups in infants. The majority of infants showed a persistent balanced profile, more than a quarter of infants had a profile that may indicate greater obesity risk, and around a fifth a profile that at baseline was largely food avoidant but showed trends of increasing food approach tendencies. Phenotype trajectory groups were related to infant formula feeding, and parent persuasive feeding practices and cognitions, but not to the trajectory of BMI *z*-score, nor parent or infant demographics. The findings provide preliminary evidence about the nature and origins of infant appetitive phenotypes and their trajectories, and therefore the possible origins of subgroup differences in appetite self-regulation in infants. Mechanistic and longer studies with sophisticated measurement of infant appetite and parent feeding are needed to further understand appetitive phenotype trajectories, their determinants, and links to dietary, health, and weight outcomes in later childhood.

## Data Availability Statement

The raw data supporting the conclusions of this article will be made available by the authors, upon reasonable request. Please note that access to the data is subject to approval by an Ethics Committee.

## Ethics Statement

The studies involving human participants were reviewed and approved by the University of Technology Sydney Human Research Ethics Committee (Ref No. 2015000528) and the Sydney Local Health District Human Research Ethics Committee (Protocol No. X15-0233) granted ethical approval for the study. The patients/participants provided their written informed consent to participate in this study.

## Author Contributions

CGR, JA, CF, EJ, CR, and ED-W designed the study. CGR, JA, CF, CR, and ED-W collected the data. AB analyzed the data with input from CGR, JA, and EJ. CGR led the writing of the article. All authors reviewed, critiqued, and approved the manuscript.

## Funding

This work was supported by the Health Futures Development Grant provided by the Faculty of Health, University of Technology, Sydney. The funder had no role in the design, analysis, or writing of this article.

## Conflict of Interest

The authors declare that the research was conducted in the absence of any commercial or financial relationships that could be construed as a potential conflict of interest.

## Publisher's Note

All claims expressed in this article are solely those of the authors and do not necessarily represent those of their affiliated organizations, or those of the publisher, the editors and the reviewers. Any product that may be evaluated in this article, or claim that may be made by its manufacturer, is not guaranteed or endorsed by the publisher.
